# Exercise rehabilitation for people with postural tachycardia syndrome at two secondary care centres in the UK: the PULSE feasibility randomised controlled trial

**DOI:** 10.1136/bmjopen-2024-090197

**Published:** 2025-02-22

**Authors:** Gordon McGregor, Becky Evans, Harbinder Kaur Sandhu, Julie Bruce, Gita Devi, Sajad Hayat, Siew Wan Hee, Peter Heine, Nikki Holliday, Shivam Joshi, Lesley Kavi, Lim Boon, Angela Noufaily, Nicholas Parsons, Shilpa Patel, Gemma Pearce, Richard Powell, Eva Schultz, Jane Simmonds, Albiona Zhupaj, Helen Eftekhari, Sandeep Panikker

**Affiliations:** 1 Coventry University, Coventry, UK; 2 University Hospitals Coventry and Warwickshire NHS Trust, Coventry, UK; 3 University of Warwick, Coventry, UK; 4 Qatar Heart Hospital, Doha, Qatar; 5 PoTS UK, London, UK; 6 Imperial College Healthcare NHS Trust, London, UK; 7 University College London, London, UK

**Keywords:** REHABILITATION MEDICINE, CARDIOLOGY, Physical Therapy Modalities

## Abstract

**Objectives:**

The aim of the study was to assess the feasibility of conducting a definitive multicentre randomised controlled trial (RCT) testing an online exercise rehabilitation and behavioural/motivational support intervention for people with postural tachycardia syndrome (PoTS).

**Design:**

Feasibility RCT.

**Setting:**

Two secondary care centres.

**Participants:**

Adults aged 18 to 60 years with PoTS. Exclusions were serious mental health/cognitive problem preventing safe participation; currently undertaking physical activity equivalent to the Chief Medical Officer guidelines; pregnancy.

**Interventions:**

Participants were randomly assigned (1:1) to best-practice usual care (a single 1:1 session of advice) or the ‘postural tachycardia syndrome exercise’ (PULSE) intervention: (1) individual online consultation, (2) 12 weeks of supervised online group exercise and behavioural/motivational support, and (3) home exercise programme with recumbent exercise bike.

**Outcomes:**

The primary outcome was feasibility: (1) patients screened, eligible, recruited, randomised, withdrawn; (2) adherence; (3) physiological, clinical and patient-reported outcomes (4 and 7 months); and (4) embedded qualitative study to evaluate acceptability.

**Results:**

209 patients screened between 5 May 2021 and 1 December 2022, 44 (female 98%; age 29.9 SD, 7.5) were randomised to usual care (n=21) or PULSE (n=23) (71% of target). Follow-up at 4 months was n=12 and n=17 respectively (66% of target). Median live exercise/support session attendance was 15 (IQR 12 to 17) of 18 sessions. Home exercise bike usage was highly variable. There were two serious adverse events in each treatment arm, both unrelated to the trial. Exercise rehabilitation was considered important by participants, and trial procedures, outcomes and interventions were acceptable.

**Conclusions:**

The PULSE trial procedures and interventions were acceptable, and important design considerations were identified. A definitive RCT testing a remotely supervised exercise rehabilitation and behavioural/motivational support intervention for people with PoTS is feasible in the UK National Health Service.

**Trial registration number:**

ISRCTN45323485.

**STRENGTHS AND LIMITATIONS OF THIS STUDY**

- The postural tachycardia syndrome exercise intervention and trial design were co-created with people affected by postural tachycardia syndrome and multiple stakeholders.
- This was a randomised trial with group allocation concealed from outcomes assessors, the principal investigator and the trial statistician.
- Feasibility was rigorously assessed with both quantitative and qualitative analyses.
- Home exercise bike usage was determined by self-report which may have been subject to bias.
- Participant interview data regarding acceptability may be skewed given these participants engaged fully with the intervention.

## Introduction

 Postural tachycardia syndrome (PoTS) is a long-term condition predominantly affecting females (>90%), typified by light-headedness, palpitations, tremulousness, cognitive dysfunction and fatigue.^1^ Activities of daily living are impaired, compounded by progressive exercise intolerance and deconditioning.^2^ Health-related quality of life (HRQoL), societal participation and economic productivity are affected.^3 4^ The coexistence of orthostatic intolerance with neurological, connective tissue, gastrointestinal and immune dysfunction means living with PoTS is challenging and pharmacological management is limited.^5^ Exercise training is often recommended as part of a multidisciplinary biopsychosocial approach to treatment.^6^

Supervised exercise rehabilitation may be an effective therapy for PoTS^2^ but safety and effectiveness have not been tested in well designed, adequately powered randomised controlled trials (RCTs).^3^ Feasibility, retrospective and prospective observational studies suggest that orthostatic intolerance, exercise capacity and psychosocial morbidity might improve with exercise-based lifestyle interventions for people with PoTS.^7^,^10^ After a semi-supervised (in-person and virtual) exercise training programme, a recent pilot RCT (n=60) reported improved aerobic fitness, orthostatic symptoms and exercise tolerance, but no overall improvement in dysautonomia symptoms measured with the Composite Autonomic Symptom Score (COMPASS 31) questionnaire.^10^ The COVID-19 pandemic accelerated the development and implementation of remotely supervised online exercise rehabilitation programmes. This mode of delivery has proven clinically effective for people with post-COVID-19 condition after hospitalisation for COVID-19.^11^ Findings may be transferrable to people living with PoTS.

Large clinical trials are needed to evaluate the effectiveness of remotely delivered exercise rehabilitation for people with PoTS. The aim of the postural tachycardia syndrome exercise (PULSE) study was to assess the feasibility of conducting a multicentre RCT testing a supervised online exercise rehabilitation and behavioural/motivational support intervention compared with best-practice usual care for people living with PoTS.

## Methods

### Study design and setting

PULSE was a two-arm feasibility RCT with embedded qualitative study recruiting from two National Health Service (NHS) PoTS clinics in England (University Hospitals Coventry & Warwickshire (UHCW) NHS Trust and Imperial College Healthcare (ICH) NHS Trust). The trial was prospectively registered (ISRCTN45323485) on 7 April 2020. The protocol and subsequent update in response to the COVID-19 pandemic are published elsewhere.^12 13^ Ethical approval was received from the East Midlands, Nottingham Research Ethics Committee (20/EM/0077) and Health Research Authority on 3 April 2020. The trial is reported in accordance with the CONSORT (Consolidated Standards of Reporting Trials) extension to pilot and feasibility trials guidelines.^14^

### Participants

Adults aged 18 to 60 years with a confirmed diagnosis of PoTS (in accordance with accepted criteria)^2^ attending specialist out-patient clinics were eligible. Inclusion criteria were willingness to attend PULSE centre three times over 7 months for outcome assessments, and access to appropriate IT infrastructure at home that is, internet and enabled device (loan device provided if required). Exclusions were serious mental health/cognitive issue that prevented engagement with trial procedures or made participation unsafe; currently undertaking structured exercise/physical activity equivalent to the Chief Medical Officer (CMO) guidelines (150 min moderate or 75 min vigorous exercise per week); pregnancy; or had taken part in the original co-creation workshops to design the PULSE intervention and trial protocol.^15^ Participants were screened from clinic lists and provided electronic informed consent.

### Randomisation and masking

After baseline outcomes assessment, participants were randomised with variable block randomisation on a 1:1 basis to intervention or usual care, stratified by centre (UHCW NHS Trust or ICH NHS Trust), using a validated online sequence generator as part of an electronic data capture system (Castor EDC). PULSE practitioners delivering the interventions, researchers conducting interviews and participants were informed of group allocation. Outcomes assessors, the principal investigator and the trial statistician were blind to group allocation.

### Interventions

#### The PULSE intervention

The PULSE intervention (online supplemental figure S1) and trial design were co-created with people with PoTS and relevant stakeholders and described in detail elsewhere.^15^ Participants attended a 1 hour, one-to-one, online consultation with a PULSE practitioner to assess medical and physical activity history and medication and to discuss goals, expectations and concerns. The PULSE practitioner prescribed and supervised an individualised 12-week exercise programme including (1) weekly live group exercise sessions delivered via Zoom (hosted on a bespoke platform www.beamfeelgood.com) allowing participants to receive real-time instruction and feedback, (2) loan of recumbent exercise bike, heart rate monitor, resistance bands and gym ball to use weekly at home, and (3) two 11 min on-demand videos of recumbent and seated exercise sessions to complete weekly at home. Behavioural and motivational support was provided weekly for 6 weeks during 1 hour online group sessions facilitated by a trained PULSE practitioner, supported by a health psychologist and a comprehensive participant workbook.

#### Control intervention: usual care

Participants in the usual care arm were directed to freely available advice on lifestyle physical activity (PoTS UK website)^16^ during an online, one-to-one consultation with a PULSE practitioner. They did not receive any further input from the PULSE team but were permitted to continue with existing physical activity, treatment and therapy.

### Outcomes

The aim of the study was to assess the feasibility of a future definitive RCT. The primary feasibility outcomes were: (1) the number of patients screened, eligible, recruited, randomised, withdrawn and retained, (2) adherence (number of sessions attended) to the supervised online exercise and behavioural and motivational support sessions, and unsupervised home exercise programme, (3) physiological, clinical and patient-reported outcomes to identify a primary outcome for a definitive trial, and (4) acceptability of the interventions and trial procedures explored within the embedded qualitative study.

### Secondary outcomes

Physiological, clinical and patient-reported outcomes were assessed at baseline, and at 4 and 7 months post-randomisation by assessors blind to group allocation. Exercise capacity was measured with a graded submaximal recumbent cycle ergometer assessment.^17^ The active stand test to assess increase in heart rate from supine to 10 min standing was conducted as per clinical practice.^18^ Dysautonomia symptom burden was measured with the COMPASS 31.^19^ This self-rating questionnaire evaluates six domains of autonomic function: orthostatic intolerance, vasomotor, secretomotor, gastrointestinal, bladder and pupillomotor domains. A total score was calculated (range 0 to 100), with a higher score indicating a greater disease burden. Other patient-reported outcome measures (PROMs) included HRQoL using the EQ-5D-5L,^20^ the Fatigue Severity Scale and the Generalised Self-Efficacy Scale.^21^ Adverse (AE) and serious adverse events (SAE) were documented in accordance with the principles of good clinical practice.^22^

### Embedded qualitative study

To explore perceptions, opinions, acceptability and experiences of trial procedures, the interventions, and outcome measures, semi-structured interviews were conducted with a sample of participants at baseline and 4 months post-randomisation. The topic guide is available in supplementary material. Interviews were digitally recorded, pseudonymised and transcribed verbatim. Data were managed with NVivo software before analysis using the framework method.^23^ Qualitative findings will be published in full elsewhere but are incorporated into the assessment of acceptability and the feasibility of conducting a future multicentre RCT.

### Sample size

This was a feasibility RCT to assess recruitment, uptake, adherence and acceptability. While the sample size was not based on a power calculation, the aim was to estimate any possible effect of the change of heart rate from supine to 10 min stand between the two trial arms using the CI approach. From a single-arm study investigating a 3-month community exercise programme in a similar population,^7^ the mean increase in heart rate from supine to 10 min stand at baseline was 46 (SD, 17) beats per minute (bpm) and post-intervention was 23 (SD, 14) bpm. The SD of the difference of the change was 17 bpm. Therefore, the total sample size required to obtain a 95% CI width of 20 bpm was 46 participants.^24^ Assuming 25% dropout, the sample size was 62 participants (31 per arm).

### Data analysis

Participant demographics and physiological, clinical and patient-reported outcomes were summarised by trial intervention arms as mean and SD, median and IQR for continuous data or frequency and percentage for categorical data at baseline, and 4 and 7 months post-randomisation. The primary outcome was feasibility; therefore, process measures relating to recruitment and intervention delivery were assessed. No formal hypothesis testing was performed. The ADePT (A Process for Decision-making after Pilot and Feasibility Trials) framework^25^ was used to identify and examine issues and problems methodically and to appraise and find appropriate solutions to inform the decision-making process to transition from a two-centre feasibility trial to a definitive multicentre RCT.

### Patient and public involvement

Patient and public involvement was integral to this study. People with PoTS were co-applicants on the grant submission and were active members of the research team. The PULSE intervention and trial design were co-created with people affected by PoTS.^15^ Two patient partners sat on the trial management group, meeting monthly for the duration of the trial. The GRIPP2 reporting checklist and three co’s framework were used throughout.^26 27^

## Results

### Recruitment

**Figure 1:**
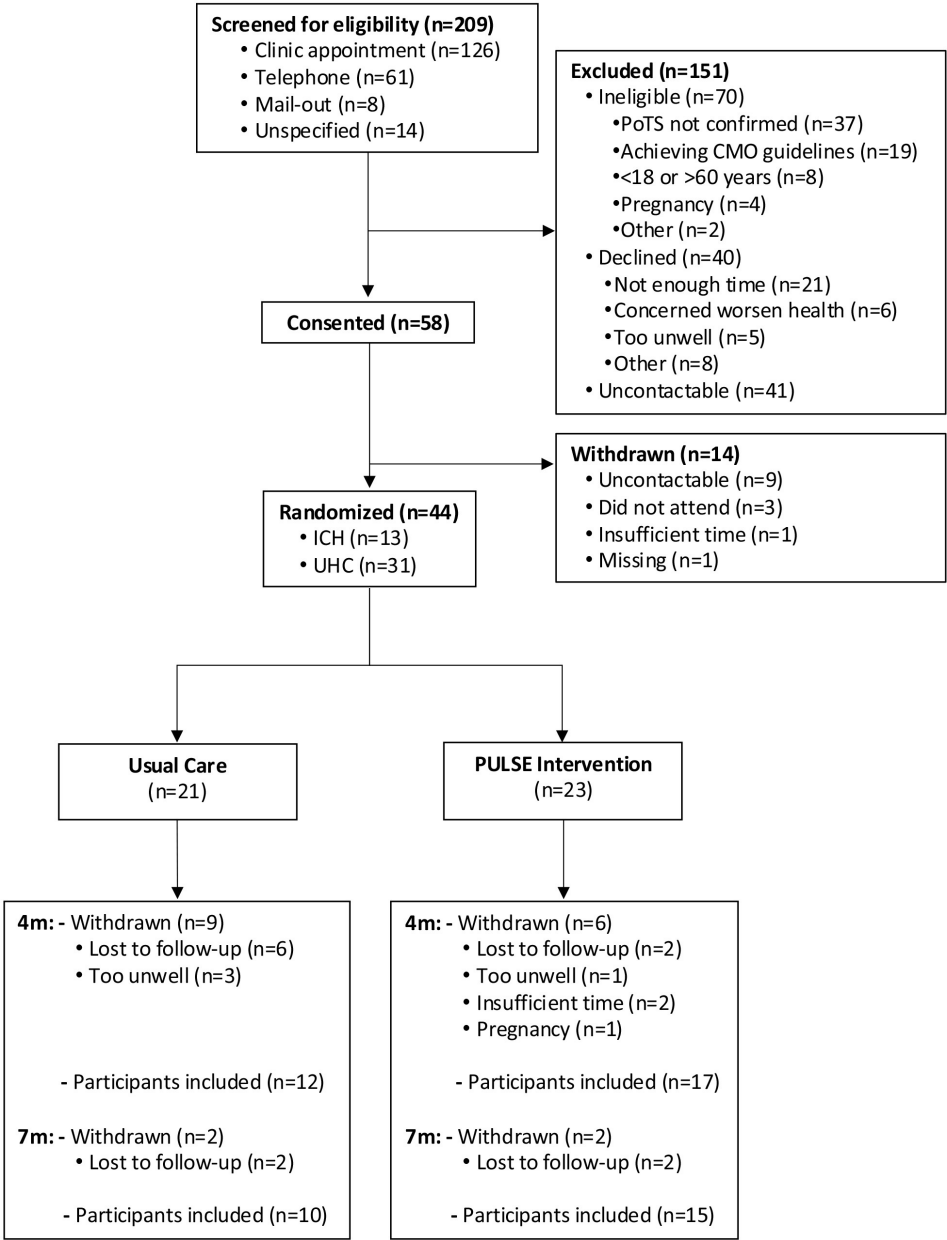
CONSORT diagram. CMO, Chief Medical Officer; CONSORT, Consolidated Standards of Reporting Trials; ICH, Imperial College Healthcare; PoTS, postural tachycardia syndrome; PULSE, postural tachycardia syndrome exercise study; UHC, University Hospitals Coventry.

A total of 209 patients were assessed for eligibility between 5 May 2021 and 1 December 2022 (figure 1). Overall, 70/209 (33%) were ineligible, 40/209 (19%) declined to take part, 41/209 (20%) were uncontactable and 58/209 (28%) consented to take part. Some participants (14/58, 24%) did not proceed to randomisation and were withdrawn. Reasons for ineligibility, declining and withdrawal are detailed in figure 1. Ultimately, 44/209 (21%) participants were randomised to either usual care (n=21) or the PULSE intervention (n=23), 31/44 (70%) from UHCW NHS Trust and 13/44 (30%) from ICH NHS Trust. We did not achieve our target of 62 participants randomised (44/62, 71%). Baseline demographic and clinical characteristics were similar between arms (table 1 and online supplemental table S1). Participants were mostly female (43/44, 98%) and of White ethnicity (40/44, 91%) with a mean age of 29.9 (SD, 7.5) years (range 18 to 55).

**Table 1:** Demographics and clinical characteristics at baseline by trial arm and overall

	Usual care(n=21)	PULSE(n=23)	All(n=44)
Age (years)	30.3 (8.6)	29.5 (6.4)	29.9 (7.5)
Height (m)	1.66 (0.05)	1.66 (0.06)	1.66 (0.06)
Weight (kg)	77.9 (18.3)	71.6 (17.5)	74.6 (17.9)
Female sex	21 (100)	22 (96)	43 (98)
Ethnicity			
White—British	19 (90)	20 (87)	39 (89)
White—Other White	1 (5)	0 (0)	1 (2)
Asian/Asian British—Indian	1 (5)	3 (13)	4 (19)
Recruitment centres			
UHCW NHS Trust	15 (71)	16 (70)	31 (70)
ICH NHS Trust	6 (29)	7 (30)	13 (30)
Comorbidities			
Hypermobility	3 (14)	0 (0)	3 (7)
Ehlers-Danlos syndrome	6 (29)	9 (39)	15 (34)
Asthma	7 (33)	4 (17)	11 (25)
Anxiety/depression	5 (24)	3 (13)	8 (18)
Migraine	2 (10)	1 (4)	3 (7)
Fibromyalgia	1 (5)	3 (13)	4 (9)
Chronic fatigue	2 (10)	1 (4)	3 (7)
Post-COVID-19 condition	0 (0)	2 (9)	2 (5)
Irritable bowel syndrome	2 (10)	7 (30)	9 (20)
Gut dysmotility	2 (10)	0 (0)	2 (5)
Gastric reflux	1 (5)	1 (4)	2 (5)
ADHD	1 (5)	1 (4)	2 (5)
Mast cell activation	2 (10)	0 (0)	2 (5)
Other	9 (43)	7 (30)	16 (60)
Medication indication			
PoTS*	29	22	51
Anxiety/depression	6 (29)	4 (17)	10 (23)
Asthma	8 (38)	1 (4)	9 (20)
Pain	4 (19)	6 (26)	10 (23)
Gastric reflux/indigestion	2 (10)	3 (13)	5 (11)
Irritable bowel syndrome	1 (5)	3 (13)	4 (9)
Other (≤2 additional indications)	11 (52)	19 (83)	26 (59)
Medication			
Salt tablets	2 (10)	4 (17)	6 (14)
Fludrocortisone	6 (29)	4 (17)	10 (23)
Ivabradine	9 (43)	6 (26)	15 (34)
Midodrine	7 (33)	4 (17)	11 (25)
Propranolol	4 (19)	3 (13)	7 (16)
Bisoprolol	1 (5)	5 (22)	6 (14)
Salbutamol	5 (24)	0 (0)	5 (11)
Sertraline	2 (10)	2 (9)	4 (9)
Amitriptyline	2 (10)	2 (9)	4 (9)
Famotidine	0 (0)	5 (22)	5 (11)
Omeprazole	3 (14)	2 (9)	5 (11)
Other (≤2 cases in total)	19 (90)	23 (100)	41 (93)

Data are mean (SD, SD) or count (percentage). Percentages are of total n in each arm.

* sSome participants were prescribed more than one medication for PoTS.

ADHD, attention deficit hyperactivity disorder; ICH NHSImperial College Healthcare National Health ServicePoTS, postural tachycardia syndromePULSEpostural tachycardia syndrome exerciseUHCW NHSUniversity Hospitals Coventry & Warwickshire National Health Service

### Adherence to interventions

Of participants randomised to the usual care arm, 20/21 (95%) attended the one-to-one online consultation. One participant withdrew between randomisation and the one-to-one consultation. In the intervention arm, 22/23 (96%) participants attended the one-to-one online consultation. Three participants withdrew between randomisation and the first group session. The median time from randomisation to the one-to-one consultation was 21 (IQR 14 to 35) days and 13 (IQR 7 to 21) days for the usual care and intervention arms respectively. 20 participants in the intervention arm were allocated to five groups (sizes: n=3, 2, 5, 4, 6 respectively), with 18/20 (90%) attending ≥4/6 support sessions and 16/20 (80%) attending ≥8/12 exercise sessions. The median number of sessions attended (exercise+support) was 15 (IQR 12 to 17) of 18 total.

18 participants used the home exercise bike 1.1 (SD, 0.9) times per week (12 weeks) for 22.4 (SD, 15.1) minutes. Nine participants used the bike less than once per week, while two used it nearly three times per week. On-demand exercise videos were viewed infrequently with the ‘week 1–6’ video viewed 19 times by 8/20 (40%) unique participants and the ‘week 7–12’ video 14 times by 2/20 (10%) participants. One participant was responsible for 11/19 and 12/14 views respectively.

### Retention

In the usual care and intervention arms respectively, 12/21 (57%) and 17/23 (78%) participants completed the 4-month follow-up; thus, we achieved 66% (29/44) of our target. Reasons for withdrawal in the usual care arm were lost to follow-up (6/9, 67%) and ‘too unwell’ (3/9, 33%). Reasons for withdrawal in the intervention arm were lost to follow-up (2/6, 33%), ‘too unwell’ (1/6, 17%), ‘insufficient time’ (2/6, 33%) and pregnancy (1/6, 17%). 7-month follow-up data were provided by 10/21 (48%) and 15/23 (65%) participants.

### Physiological, clinical and patient-reported outcomes

Table 2 shows outcomes by treatment arm at baseline, 4-month and 7-month follow-up. Estimates for the adjusted mean difference in outcomes between the usual care and intervention arms at 4-month and 7-month follow-up are available in online supplemental table S3. The study was not statistically powered to detect a treatment effect in any of the secondary outcomes; thus, none can be proposed. However, the total time completed on the cycle ergometer test was greater in the intervention arm compared with usual care arm at the 4-month (7 (2.7) vs 6 (1.7) mins) and 7-month (8.6 (3.2) vs 8.0 (1.4) mins) follow-up. Data from PROMs may suggest a favourable effect of the PULSE intervention for example, symptoms as measured with the COMPASS 31 reduced more from baseline in the intervention arm compared to the usual care arm at the 4-month (−6 points vs +1 point) and 7-month (−9 points vs −2 points) follow-ups. The mean completion time for the COMPASS 31 questionnaire was 5.1 (SD, 2.4) minutes.

**Table 2:** Summary of outcomes by treatment arm at baseline, 4-month and 7-month follow-up

	Usual care			PULSE intervention		
	Baseline(n=21)	4-month(n=12)	7-month(n=10)	Baseline(n=23)	4-month(n=17)	7-month(n=15)
Active stand test						
Total time (minutes) (higher value=better)	8.3 (2.6)	9.4 (1.9)	10 (0.0)	8.3 (3.0)	8.8 (2.3)	9.4 (1.1)
Increase in heart rate (bpm) (lower change=better)	22.0 (8.8)	22.0 (11.0)	20.0 (9.9)	22.0 (12.0)	18.8 (10.2)	22.5 (12.1)
Graded recumbent cycle ergometer test						
Total time (minutes) (higher value=better)	6.2 (1.9)	6 (1.7)	8 (1.4)	5.9 (2.7)	7 (2.7)	8.6 (3.2)
Maximum watts (higher value=better)	55 (17)	57 (20)	69 (13)	50 (19)	57 (22)	67 (18)
COMPASS 31 dysautonomia questionnaire (range 0 to 100; higher value=more severe)	48 (8)	49 (14)	46 (11)	53 (12)	47 (14)	44 (12)
Fatigue Severity Scale (range 1 to 7; higher value=more fatigue)	5.9 (0.9)	5.9 (0.9)	6.2 (0.7)	6.3 (0.7)	5.5 (1.1)	5.6 (0.9)
General Self-Efficacy Scale (range 10 to 40; higher value=more self-efficacy)	29.0 (4.7)	29.0 (4.7)	28.0 (3.5)	27.0 (6.3)	29.0 (4.8)	29.0 (4.4)
EQ-5D-5L (range 0 to 1; higher value=better quality of life)	0.57 (0.20)	0.46 (0.21)	0.48 (0.19)	0.36 (0.28)	0.53 (0.21)	0.57 (0.24)
EQ-5D-5L Visual Analogue Scale (range 0 to 100; higher value=better health)	55 (14)	49 (19)	56 (16)	45 (14)	56 (17)	59 (19)

Data as mean (SD).

bpm, beats per minute; COMPASS, Composite Autonomic Symptom ScorePULSEpostural tachycardia syndrome exercise

### Acceptability

We interviewed 27 participants at baseline (29.9 (SD, 8.0) years), 13 (8 usual care, 5 intervention) at 4-month follow-up and eight (2 usual care, 3 intervention, 3 not randomised) participants who dropped out. For our assessment of acceptability and feasibility, we identified three key themes.

*Importance of exercise:* most participants reported that exercise was important for people with PoTS (and important to them) but many found it difficult to commit regularly due to symptoms prohibiting participation, motivation and concern that their condition would be exacerbated, and overall health negatively impacted. The desire to return to the levels of physical activity tolerated before PoTS diagnosis was a key motivation to take part in the trial.

*Trial experience and views on feasibility:* lack of time and poor health were barriers to engagement with the intervention along with some logistical issues for example, not enough space for the exercise bike at home and some live online session technical difficulties. Generally, participants were disappointed about control group allocation but were not deterred from participation; randomisation, therefore, seemed acceptable. Having the exercise bike at home was considered incredibly important. It provided a flexible, time-efficient exercise option and was seen as a real motivator; a common theme was the disappointment/loss when the bike was returned to the trial team. Live exercise and support sessions were very well received.

*Perceived trial effectiveness:* participants consistently reported that initial fears of symptom exacerbation proved to be unfounded. Temporary worsening of symptoms after exercise, which resolved quickly, was very common. Perceived improvements in physical and mental health were mixed. Some participants noticed big improvements in their activity tolerance and emotional well-being, while others were not aware of any discernible change. No participants reported deterioration in their physical or mental health.

### Adverse events

There were four SAEs (two in each arm), all of which were unrelated to the interventions or trial procedures (table 3). The two SAEs in the usual care arm were due to hospitalisation for severe upper abdominal pain and left leg nerve pain respectively. The two SAEs in the intervention arm were due to hospitalisation for chest pain and shortness of breath, and a diagnosis of fibromyalgia respectively. The intervention was well tolerated but there were more AEs reported in the intervention arm compared with usual care (34 vs 15) (table 3). In the usual care arm, 1/15 (7%) AEs were definitely related to trial procedures vs 3/34 (9%) in the intervention arm.

**Table 3:** Relatedness of serious adverse events (SAE) and adverse events (AE) by trial arm and overall

	Usual care (n=21)	PULSE (n=23)	All (n=44)
SAEs			
Related	0	0	0
Unrelated	2	2	4
**Total**	**2**	**2**	**4**
AEs			
Definitely	1	3	4
Probably	1	6	7
Possibly	4	7	11
Unlikely	0	1	1
Unrelated	9	17	26
Total*	**15**	**34**	**49**

Values are counts.

* Number of events may exceed the total number of participants in that arm because participants may have multiple events.

PULSEpostural tachycardia syndrome exercise

## Discussion

The PULSE study found that trial procedures and interventions were acceptable to participants. Data from process measures identified important design considerations and confirmed that a future definitive RCT is feasible.

Of 209 patients screened at two secondary care clinics, 21% were randomised. While this conversion rate is acceptable, it is likely under-representative of what could be achieved in a future RCT. More than half of ineligible patients did not have a confirmed PoTS diagnosis. This was due to the screening of general cardiac arrhythmia clinic patient lists at one study site rather than an exclusively PoTS patient lists. Future screening strategies should focus only on those with a confirmed diagnosis. A further 27% were ineligible due to reportedly achieving CMO physical activity guidelines. Self-reported physical activity is often unreliable^28^ and given that 40% of the general population do not achieve CMO guidelines,^29^ it is unlikely that 27% of the screened patients with PoTS were achieving these recommendations. While the intervention may be of limited benefit to those who are highly active, future eligibility criteria should be more lenient to account for over-reporting, thus, including those who are low or moderately active and may benefit.

We did not achieve our target of randomising 62 participants (71%) primarily due to the COVID-19 pandemic which severely disrupted the recruitment period and resulted in trial and intervention redesign.^13 15^ Research capacity within the NHS was reduced meaning screening and recruitment were hindered. Equally, patients were reluctant to engage in research given the risk of COVID-19 infection in a clinically vulnerable population. Lack of time was a commonly cited barrier to participation, indicating a future trial design should carefully consider time efficiency, flexibility and convenience. We achieved 66% of our 4-month follow-up target (34% vs 25% dropout) indicating that sample size estimates for a future RCT should account for a higher dropout rate.

Adherence to supervised live online exercise and support sessions was excellent (median 15 (IQR 12 to 17) of 18 sessions) and participants reported high acceptability. Unsupervised home exercise bike usage was variable; however, participant interviews showed that the bike was very important to people as it provided excellent convenience and flexibility while serving as a constant reminder and motivator to take part. Participants were very disappointed to return bikes at the end of the trial. The logistics of providing home exercise bikes were at times challenging, but given the apparent importance of this modality, it is likely a key intervention component for a future trial. On-demand home exercise videos were not routinely used and may not be a necessary component. The intervention was well tolerated with only 1/15 (7%) and 3/34 (9%) AEs definitely related to trial procedures in the usual care and intervention arms respectively. Higher AE reporting (15 vs 34) in the intervention arm was likely due to the regular contact between participants and PULSE practitioners.

The PULSE trial was not designed to detect any treatment effects. Secondary clinical, physiological and patient-reported outcomes were included only to assess feasibility and identify a candidate primary outcome for a future RCT. With no sample size calculation or hypothesis testing, findings are illustrative only. Outcome measures were mostly deemed acceptable; however, the in-person physical function tests (exercise bike and active stand tests) were less well received than the online PROMs due to the inconvenience and time required to physically attend a research facility, and the perception (and in some cases the eventuality) that testing would lead to symptom exacerbation. A remotely administered PROM may be the most appropriate candidate for a primary outcome in a future RCT. The disease-specific COMPASS 31 questionnaire may be suitable as it took on average 5.1 (SD, 2.4) minutes to complete and there was some indication of a favourable effect in the intervention arm compared with usual care at the 4-month and 7-month follow-ups. Participant report of perceived benefit was heterogenous in terms of exercise tolerance, dysautonomia symptoms and emotional well-being. Importantly, while some short-term exacerbation of symptoms post-exercise was at times evident, there were no reports of the intervention being detrimental to overall health.

### Strengths and limitations

PULSE was a feasibility RCT with insufficient statistical power to assess any treatment effect; therefore, results should be interpreted with reference to the feasibility of conducting a future definitive RCT only. While we objectively measured attendance at live exercise and support sessions, we were reliant on self-report for home exercise bike usage. Self-report of physical activity is often unreliable; thus, these data should be viewed with caution. A future study should consider alternative methods for collecting these data. We interviewed five participants who had completed the PULSE intervention. Views expressed regarding acceptability are likely skewed given these participants engaged fully with the intervention. However, we also interviewed eight participants who dropped out (three from intervention) to increase the likelihood of a diverse and representative perspective.

### Conclusions

The PULSE intervention and trial procedures were acceptable and, with acknowledgement of several important design considerations, a definitive RCT is feasible in the UK NHS. Findings support proceeding to test the clinical and cost effectiveness of this remotely supervised online exercise rehabilitation and behavioural/motivational support intervention for people with PoTS.

## supplementary material

10.1136/bmjopen-2024-090197 online supplemental file 1

## Data Availability

Data are available upon reasonable request.

## References

[1] Shaw BH, Stiles LE, Bourne K (2019). The face of postural tachycardia syndrome - insights from a large cross-sectional online community-based survey. J Intern Med.

[2] Raj SR, Guzman JC, Harvey P (2020). Canadian Cardiovascular Society Position Statement on Postural Orthostatic Tachycardia Syndrome (POTS) and Related Disorders of Chronic Orthostatic Intolerance. Can J Cardiol.

[3] Raj SR, Bourne KM, Stiles LE (2021). Postural orthostatic tachycardia syndrome (POTS): Priorities for POTS care and research from a 2019 National Institutes of Health Expert Consensus Meeting – Part 2. Auton Neurosci.

[4] Seeley M-C, Gallagher C, Ong E (2023). Poor health-related quality of life in postural orthostatic tachycardia syndrome in comparison with a sex- and age-matched normative population. Clin Auton Res.

[5] Peebles KC, Jacobs C, Makaroff L (2024). The use and effectiveness of exercise for managing postural orthostatic tachycardia syndrome in young adults with joint hypermobility and related conditions: A scoping review. Auton Neurosci.

[6] Vernino S, Bourne KM, Stiles LE (2021). Postural orthostatic tachycardia syndrome (POTS): State of the science and clinical care from a 2019 National Institutes of Health Expert Consensus Meeting - Part 1. Auton Neurosci.

[7] George SA, Bivens TB, Howden EJ (2016). The international POTS registry: Evaluating the efficacy of an exercise training intervention in a community setting. Heart Rhythm.

[8] Gibbons CH, Silva G, Freeman R (2021). Cardiovascular exercise as a treatment of postural orthostatic tachycardia syndrome: A pragmatic treatment trial. Heart Rhythm.

[9] Svensson A, Svensson-Raskh A, Holmström L (2024). Individually tailored exercise in patients with postural orthostatic tachycardia syndrome related to post-COVID-19 condition - a feasibility study. Sci Rep.

[10] Wheatley-Guy CM, Shea MG, Parks JK (2023). Semi-supervised exercise training program more effective for individuals with postural orthostatic tachycardia syndrome in randomized controlled trial. Clin Auton Res.

[11] McGregor G, Sandhu H, Bruce J (2024). Clinical effectiveness of an online supervised group physical and mental health rehabilitation programme for adults with post-covid-19 condition (REGAIN study): multicentre randomised controlled trial. BMJ.

[12] McGregor G, Hee SW, Eftekhari H (2020). Protocol for a randomised controlled feasibility trial of exercise rehabilitation for people with postural tachycardia syndrome: the PULSE study. Pilot Feasibility Stud.

[13] McGregor G, Evans B, Sandhu H (2022). Protocol update for a randomised controlled feasibility trial of exercise rehabilitation for people with postural tachycardia syndrome: the PULSE study. Pilot Feasibility Stud.

[14] Eldridge SM, Chan CL, Campbell MJ (2016). CONSORT 2010 statement: extension to randomised pilot and feasibility trials. Pilot Feasibility Stud.

[15] Pearce G, Holliday N, Sandhu H (2023). Co-creation of a complex, multicomponent rehabilitation intervention and feasibility trial protocol for the PostUraL tachycardia Syndrome Exercise (PULSE) study. Pilot Feasibility Stud.

[16] POTS UK Managing PoTS.

[17] ACSM (2022). Guidelines for exercise testing and prescription, 10th edn.

[18] Bryarly M, Phillips LT, Fu Q (2019). Postural Orthostatic Tachycardia Syndrome: JACC Focus Seminar. J Am Coll Cardiol.

[19] Sletten DM, Suarez GA, Low PA (2012). COMPASS 31: a refined and abbreviated Composite Autonomic Symptom Score. Mayo Clin Proc.

[20] Herdman M, Gudex C, Lloyd A (2011). Development and preliminary testing of the new five-level version of EQ-5D (EQ-5D-5L). Qual Life Res.

[21] Schwarzer RJM (1995). Measures in health psychology: a user’s portfolio. Causal and control beliefs.

[22] International Council for Harmonisation Integrated addendum to ICH E6 (R1): guideline for good clinical practice.

[23] Gale NK, Heath G, Cameron E (2013). Using the framework method for the analysis of qualitative data in multi-disciplinary health research. BMC Med Res Methodol.

[24] Machin D, Campbell M, Tan S (2018). Sample sizes for clinical, laboratory and epidemiological studies, 4th edn.

[25] Bugge C, Williams B, Hagen S (2013). A process for Decision-making after Pilot and feasibility Trials (ADePT): development following a feasibility study of a complex intervention for pelvic organ prolapse. Trials.

[26] Staniszewska S, Brett J, Simera I (2017). GRIPP2 reporting checklists: tools to improve reporting of patient and public involvement in research. BMJ.

[27] Pearce G, Magee P (2024). Co-creation solutions and the three Co’s framework for applying Co-creation.

[28] Brenner PS, Lies DJ, Lies D (2016). Identity as a cause of measurement bias. Soc Psychol Q.

[29] Sport England (2023). Active Lives Adult Survey November 2021-22 Report. Department for Digital, Culture, Media & Sport, HM Government.

