# Supplementary material for: Exercise rehabilitation for people with postural tachycardia syndrome at two secondary care centres in the UK: the PULSE feasibility randomised controlled trial
Source: BMJ Open. 2025 Feb 22;15(2):e090197. doi: 10.1136/bmjopen-2024-090197 (PMC11848665; doi:10.1136/bmjopen-2024-090197)
Supplement: online supplemental file 1 [file bmjopen-15-2-s001.docx]

**Exercise rehabilitation for people with postural tachycardia syndrome: the PULSE feasibility randomised controlled trial.**

**Supplementary material**

**Figure S1.** The PULSE intervention


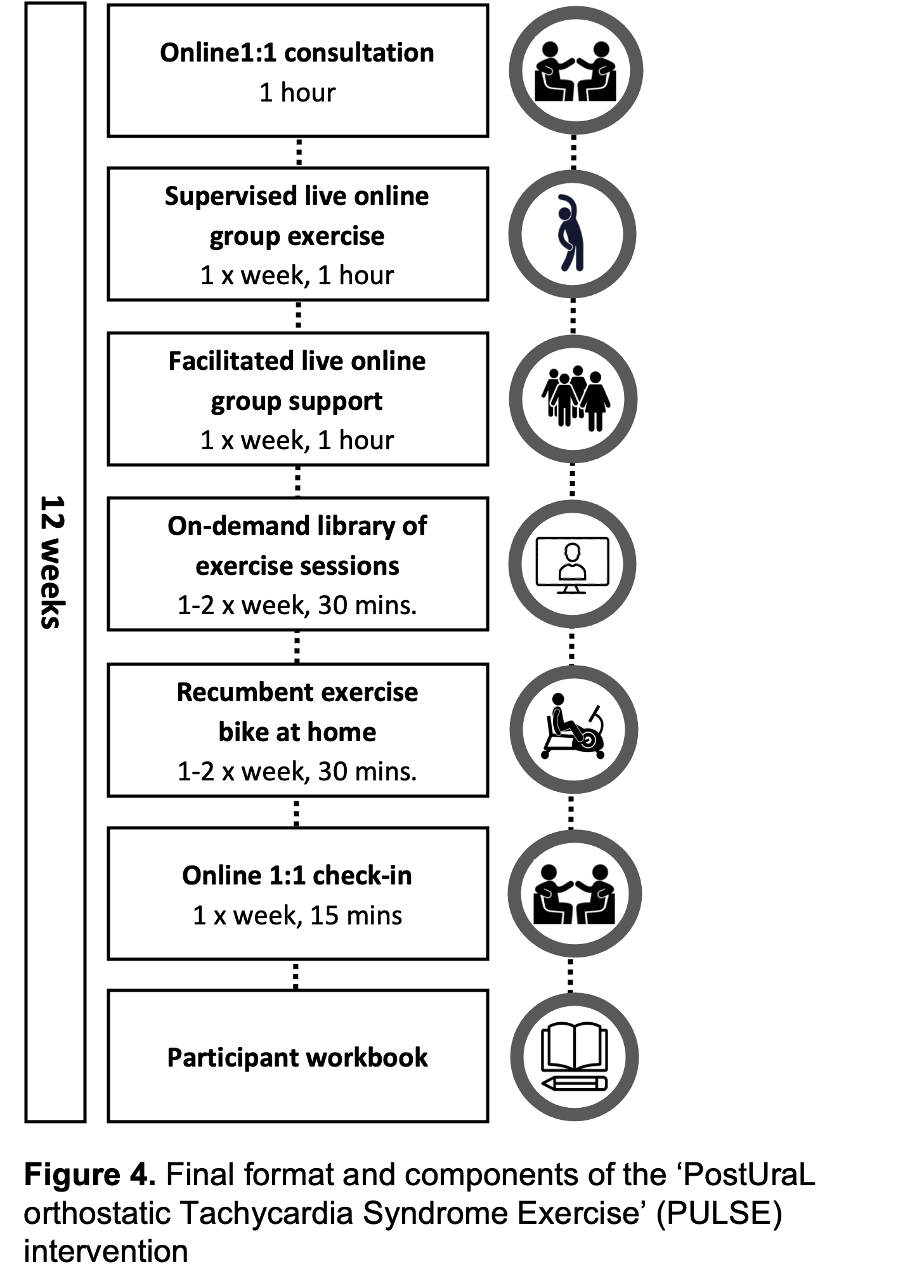


**Table S1.** Baseline characteristics by trial arm

| **Characteristics** | **Usual care**  **(N=21)** | **PULSE**  **(N=23)** | **All**  **(N=44)** |
| --- | --- | --- | --- |
| **Age (years)** |  |  |  |
| N |  |  |  |
| Mean (SD) | 30.3 (8.6) | 29.5 (6.4) | 29.9 (7.5) |
| Median (IQR) | 28.0 (26,33) | 29 (25,33) | 28 (25,33) |
| Min to Max | 18-55 | 20-51 | 18-55 |
| **Missing** | 5 |  |  |
| **Sex** |  |  |  |
| Female | 21 | 22 | 43 |
| Male | 0 | 1 | 1 |
| **Ethnicity** |  |  |  |
| White - British | 19 | 20 | 39 |
| White - Other White | 1 | 0 | 1 |
| Asian/Asian British - Indian | 1 | 3 | 4 |
| **Height (m)** |  |  |  |
| Mean (SD) | 1.66 (0.05) | 1.66 (0.06) | 1.66 (0.06) |
| Median (IQR) | 1.66 (1.63,1.67) | 1.67 (1.63,1.70) | 1.66 (1.63,1.69) |
| Min to Max | 1.57-1.75 | 1.54-1.77 | 1.54-1.77 |
| **Weight (kg)** |  |  |  |
| Mean (SD) | 77.9 (18.3) | 71.6 (17.5) | 74.6 (17.9) |
| Median (IQR) | 76.3 (62.2,87.0) | 69.9 (57.2,79.6) | 71.6 (60.2,87.0) |
| Min to Max | 48.4-120.0 | 49.0-113.0 | 48.4-120.0 |

Data as number (percent), mean (standard deviation) or median (inter-quartile range). Abbreviations: SD, standard deviation; IQR, interquartile range.

**Table S2.** Summary of outcomes by treatment arms at baseline, 4- and 7-month follow-up

| **Outcomes** | **Usual care** | | | **PULSE intervention** | | | **All** | | |
| --- | --- | --- | --- | --- | --- | --- | --- | --- | --- |
|  | **Baseline**  **(N = 21)** | **4-month**  **(N =12)** | **7-month**  **(N =10)** | **Baseline**  **(N =23)** | **4-month**  **(N = 17)** | **7-month**  **(N = 15)** | **Baseline**  **(N = 44)** | **4-month**  **(N =29)** | **7-month**  **(N =25)** |
|  | | | | | | | | | |
| **Active stand test** | | | | | | | | | |
| Resting heart rate (bpm) [lower value = better] | | | | | | | | | |
| N | 21 | 10 | 8 | 23 | 17 | 11 | 44 | 27 | 19 |
| Mean (SD) | 78(18) | 76(20) | 71(13) | 79(14) | 78(10) | 76(8) | 79(16) | 78(14) | 74(10) |
| Median (IQR) | 78(66,86) | 76(62,83) | 67(62,78) | 81(69,89) | 79(75,85) | 76(70,82) | 78(66,89) | 79(69,85) | 75(65,82) |
| Min to Max | 43-118 | 50-117 | 56-92 | 57-103 | 57-93 | 63-85 | 43-118 | 50-117 | 56-92 |
| Maximum heart rate (bpm) [lower value = better] | | | | | | | | | |
| N | 21 | 10 | 8 | 23 | 17 | 11 | 44 | 27 | 19 |
| Mean (SD) | 101(21) | 96(21) | 93(16) | 100(13) | 99(19) | 98(16) | 101(17) | 98(19) | 96(16) |
| Median (IQR) | 107(86,109) | 100(80,108) | 92(85,101) | 101(95,107) | 98(86,110) | 91(88,112) | 104(87,109) | 99(84,110) | 91(87,108) |
| Min to Max | 54-136 | 61-132 | 71-122 | 73-126 | 75-135 | 73-122 | 54-136 | 61-135 | 71-122 |
| Total time (minutes) [higher value = better] | | | | | | | | | |
| N | 21 | 10 | 8 | 23 | 17 | 11 | 44 | 27 | 19 |
| Mean (SD) | 8.3(2.6) | 9.4(1.9) | 10(0) | 8.3(3) | 8.8(2.3) | 9.4(1.1) | 8.3(2.7) | 9(2.1) | 9.7(0.9) |
| Median (IQR) | 10(7,10) | 10(10,10) | 10(10,10) | 10(7.5,10) | 10(8,10) | 10(9.6,10) | 10(7,10) | 10(10,10) | 10(10,10) |
| Min to Max | 1.6-10 | 4-10 | 10-10 | 1-10 | 2.4-10 | 7-10 | 1-10 | 2.4-10 | 7-10 |
| Increase in heart rate at 10 minutes or total completed time (bpm) [lower change = better] | | | | | | | | | |
| N | 21 | 10 | 8 | 23 | 17 | 11 | 44 | 27 | 19 |
| Mean (SD) | 22(8.8) | 22(11) | 20(9.9) | 22(12) | 18.8(10.2) | 22.5(12.1) | 22(11) | 20(10.5) | 21.4(11) |
| Median (IQR) | 24(17,28) | 22(17,30) | 18(13,25) | 22(12,29) | 19(12,25) | 24(12,32) | 23(15,28) | 20(13,26) | 21(12,31) |
| Min to Max | 7-40 | 1-39 | 8-35 | -2-44 | 0-45 | 6-41 | -2-44 | 0-45 | 6-41 |
|  | | | | | | | | | |
| **Graded recumbent cycle ergometer test** | | | | | | | | | |
| Resting heart rate (bpm) [lower value = better] | | | | | | | | | |
| N | 20 | 9 | 8 | 23 | 16 | 11 | 43 | 25 | 19 |
| Mean (SD) | 80(18) | 74(14) | 71(13) | 79(12) | 77(16) | 75(7) | 80(15) | 76(12) | 73(10) |
| Median (IQR) | 78 (66,90) | 78(62,83) | 67(62,78) | 76(73,89) | 77(69,89) | 76(70,81) | 78(71,89) | 78(68,89) | 76(65,81) |
| Min to Max | 50-122 | 57-97 | 56-92 | 59-107 | 57-91 | 63-85 | 50-122 | 57-97 | 56-92 |
| Maximum heart rate (bpm) [lower value = better] | | | | | | | | | |
| N | 20 | 9 | 8 | 23 | 16 | 11 | 43 | 25 | 19 |
| Mean (SD) | 111(18) | 109(16) | 120(21) | 108(18) | 112(16) | 120(19) | 109(18) | 111(16) | 120(19) |
| Median (IQR) | 112(104,124) | 114(104,122) | 123(119,128) | 104(98,117) | 109(101,118) | 116(111,124) | 110(98,119) | 110(101,122) | 119(113,128) |
| Min to Max | 78-138 | 82-127 | 74-148 | 89-174 | 86-154 | 95-156 | 78-174 | 82-154 | 74-156 |
| Total time (minutes) [higher value = better] | | | | | | | | | |
| N | 20 | 9 | 8 | 23 | 16 | 11 | 43 | 25 | 19 |
| Mean (SD) | 6.2(1.9) | 6(1.7) | 8(1.4) | 5.9(2.7) | 7(2.7) | 8.6(3.2) | 6(2.4) | 7(2) | 8.4(2.6) |
| Median (IQR) | 6(5,7) | 7(6,7) | 8(7,8.5) | 5(4.5,7) | 7(5,8) | 8(6,9.5) | 6(5,7) | 7(5,8) | 8(6.5,9.5) |
| Min to Max | 3-12 | 3-8 | 6-10 | 2-16 | 3-14 | 6-15 | 2-16 | 3-14 | 6-15 |
| Maximum watts [higher value = better] | | | | | | | | | |
| N | 20 | 9 | 8 | 23 | 16 | 11 | 43 | 25 | 19 |
| Mean (SD) | 55(17) | 57(20) | 69(13) | 50(19) | 57(22) | 67(18) | 52(18) | 57(21) | 68(16) |
| Median (IQR) | 52(43,64) | 65(43,69) | 70(69,75) | 51(38,61) | 62(47,72) | 68(59,82) | 51(40,62) | 64(43,70) | 70(65,81) |
| Min to Max | 25-90 | 30-82 | 40-82 | 10-90 | 10-85 | 30-90 | 10-90 | 10-85 | 30-90 |
| Termination reason graded recumbent cycle ergometer test | | | | | | | | | |
| Dizziness/pre-syncope | 1 | 0 | 0 | 0 | 0 | 0 | 1 | 0 | 0 |
| General Fatigue | 19 | 9 | 7 | 19 | 13 | 9 | 38 | 22 | 16 |
| Peripheral fatigue | 0 | 0 | 0 | 0 | 1 | 0 | 0 | 1 | 0 |
| Chest pain | 0 | 0 | 0 | 0 | 0 | 1 | 0 | 0 | 1 |
| Joint pain | 0 | 0 | 0 | 1 | 2 | 0 | 1 | 2 | 0 |
| Unable to maintain cadence | 0 | 0 | 0 | 2 | 0 | 0 | 2 | 0 | 0 |
| Other | 0 | 0 | 1 | 1 | 0 | 1 | 1 | 0 | 2 |
| List of other reasons to terminate recumbent cycle ergometer test | | | | | | | | | |
| Headache/migraine | 0 | 0 | 0 | 0 | 0 | 1 | 0 | 0 | 1 |
| Difficulty pedalling | 0 | 0 | 1 | 1 | 0 | 0 | 1 | 0 | 1 |
|  | | | | | | | | | |
| **Patient reported outcomes** | | | | | | | | | |
| COMPASS 31 dysautonomia questionnaire (range, 0 to 100; higher more severe) | | | | | | | | | |
| N | 16 | 15 | 7 | 19 | 14 | 11 | 35 | 29 | 18 |
| Mean (SD) | 48(8) | 49(14) | 46(11) | 53(12) | 47(14) | 44(12) | 50(10) | 48(14) | 45(12) |
| Median (IQR) | 50(41,54) | 45(38,60) | 41(38,54) | 54(45,59) | 46(38,57) | 42(36,50) | 52(41,59) | 45(38,58) | 42(37,50) |
| Min to Max | 36-60 | 32-75 | 37-62 | 33-81 | 26-68 | 23-64 | 33-81 | 26-75 | 23-64 |
| Fatigue severity scale (FSS) (range, 1 to 7; higher more fatigue) | | | | | | | | | |
| N | 23 | 25 | 16 | 24 | 28 | 16 | 47 | 53 | 32 |
| Mean (SD) | 5.9(0.9) | 5.9(0.9) | 6.2(0.7) | 6.3(0.7) | 5.5(1.1) | 5.6(0.9) | 6.1(0.8) | 5.7(1) | 5.8(0.8) |
| Median (IQR) | 6.1(5.1,6.3) | 5.9(5.3,6.7) | 6.3(5.9,6.6) | 6.4(5.9,7) | 5.4(5,6.4) | 5.7(5.2,6.2) | 6.1(5.8,6.8) | 5.8(5,6.6) | 5.9(5.4,6.6) |
| Min to Max | 4-7 | 4.2-7 | 4.9-6.9 | 4.6-7 | 3.3-7 | 3.9-7 | 4-7 | 3.3-7 | 3.9-7 |
| General self-efficacy scale (range, 10 to 40; higher more self-efficacy) | | | | | | | | | |
| N | 23 | 25 | 16 | 24 | 28 | 16 | 47 | 53 | 32 |
| Mean (SD) | 29(4.7) | 29(4.7) | 28(3.5) | 27(6.3) | 29(4.8) | 29(4.4) | 28(5.7) | 29(4.7) | 29(4.0) |
| Median (IQR) | 29(2.7,31) | 28(25,32) | 28(27,30) | 27(24,31) | 30(26,31) | 30(28,31) | 29(25,31) | 29(26,32) | 29(27,31) |
| Min to Max | 18-38 | 22-39 | 23-34 | 13-37 | 16-37 | 18-35 | 13-38 | 16-39 | 18-35 |
| EQ-5D-5L (range, 0 to 1; higher better quality of life) | | | | | | | | | |
| N | 18 | 15 | 7 | 19 | 18 | 11 | 37 | 33 | 18 |
| Mean (SD) | 0.57(0.20) | 0.46(0.21) | 0.48(0.19) | 0.36(0.28) | 0.53(0.21) | 0.57(0.24) | 0.46(0.27) | 0.50(0.21) | 0.53(0.22) |
| Median (IQR) | 0.61(0.43,0.67) | 0.54(0.31,0.59) | 0.41(0.32,0.61) | 0.41(0.18,0.57) | 0.54(0.41,0.71) | 0.64(0.57,0.69) | 0.55(0.32,0.64) | 0.54(0.33,0.65) | 0.60(0.40,0.69) |
| Min to Max | 0.15-0.91 | 0.10-0.80 | 0.30-0.79 | -0.22-0.74 | 0.12-0.84 | -0.08-0.74 | -0.21-0.91 | 0.10-0.84 | -0.08-0.79 |
| EQ-5D-5L VAS (range, 0 to 100; higher better health) | | | | | | | | | |
| N | 18 | 15 | 7 | 19 | 18 | 11 | 37 | 33 | 18 |
| Mean (SD) | 55(14) | 49(19) | 56(16) | 45(14) | 56(17) | 59(19) | 50(15) | 53(18) | 58(17) |
| Median (IQR) | 50(42,64) | 45(40,65) | 60(46,70) | 40(39,48) | 54(49,64) | 60(46,70) | 45(40,58) | 50(40,65) | 60(43,70) |
| Min to Max | 35-82 | 19-80 | 30-70 | 20-80 | 30-90 | 30-90 | 20-82 | 19-90 | 30-90 |

Data as number (percent), mean (standard deviation) or median (inter-quartile range). Abbreviations: SD, standard deviation; IQR, interquartile range; VAS, visual analogue scale.

**Table S3.** Difference in outcomes between trial arms at 4- and 7-month follow-ups. Data as mean (95% confidence interval, CI) difference between arms using a generalised linear model adjusted for baseline values and age. **A positive difference indicates that the PULSE intervention may be superior.**

|  | **4-month** | **7-month** |
| --- | --- | --- |
|  | **Estimates (95%CI)** | **Estimates (95%CI)** |
| **Active stand test** |  |  |
| Total time (minutes) | -0.3 (-1.8 to 1.1) | -0.6 (-1.5 to 0.3) |
| Increase in heart rate (bpm) | 2.1 (-5.6 to 9.7) | -3.8 (-13.2 to 5.5) |
| **Graded cycle ergometer test** |  |  |
| Total time (minutes) | 0.5 (-1.4 to 2.5) | 0.4 (-1.6 to 2.4) |
| Maximum workload (Watts) | 2.9 (-9.2 to 15.0) | 1.6 (-11.6 to 14.9) |
| **Patient reported outcomes** |  |  |
| COMPASS 31 *(range, 0 to 100)* | 4.0 (-6.2 to 14.2) | 5.7 (-5.2 to 16.6) |
| Fatigue severity scale *(range, 1 to 7)* | 0.61 (0.13 to 1.10) | 0.57 (-0.28 to 1.42) |
| General self-efficacy scale *(range, 10 to 40)* | 0.5 (-2.6 to 3.6) | 0.8 (-2.8 to 4.3) |
| EQ-5D-5L *(range, -0.285 to 1)* | 0.14 (-0.03 to 0.31) | 0.21 (0.01 to 0.41) |
| EQ-5D-5L VAS *(range, 0 to 100)* | 12.0 (0.89 to 23.1) | 8.2 (-9.6 to 26.0) |

**PULSE qualitative interview schedules/topic guide**

Aim of the PULSE project: The aim of the overall trial is to assess the feasibility of a supervised exercise rehabilitation intervention with behavioural and motivational support for people with POTS.

Aim of the qualitative analysis: To explore perceptions, opinions, acceptability and experiences of trial procedures, the PULSE intervention, and outcome measures, we will interview a sample of participants, drop-outs, and those who declined participation.

Pre-trial (participants)

1. How did you find out about the trial?
2. Did you have any questions prior to taking part in the trial?
   1. Were these answered satisfactorily?
3. What made you want to take part?
4. What did you think of the Participant Information Sheet you were given? [provide example to jog memory]
   1. Was it clear enough?
   2. What would you change?
   3. What would you keep the same?
5. What are you hoping to achieve by taking part in the research?
6. How important is exercise to you?
7. Do you have any expectations about whether this intervention might work for you?
8. Did you have any expectations about what group you would be in?
9. Do you have any concerns about taking part in the trial?
10. Do you have any other comments you would like to add?

Post-trial (participants)

Qs on intervention

1. What did you feel about being allocated to the group you were in?
2. What did you think of the online activity sessions?
3. What made it easy to take part the online sessions?
4. What made it difficult to take part in online sessions?
5. What did you think of doing the activities at home?
6. What made it easy to take part the in activities at home?
7. What made it difficult to take part in the activities at home in your everyday life?
8. Tell me about how easy or difficult it was to fit the at home activities around your daily activities?
9. Did you experience any symptoms when completing the activities?
10. Did your symptoms impact your ability to complete the home activities?
11. Did you feel safe completing the activities at home?
12. Were the exercises easy to learn?
13. What did you think of your practitioner who ran the sessions?

Qs on outcome measures

Bring examples of outcome measures to interview to show face to face/on screen/or email beforehand if telephone interview so they participant knows what specific outcome measure is being discussed

1. How did you feel completing the 10 minute stand test?
   1. Do you think it was an important part of the research?
   2. Would you want this measure to be included in future trials?
2. What did you feel about the number of questionnaires you had to complete?
3. What did you think about the time it took to complete those questionnaires?
4. How did you feel completing the [COMPASS scale – or however it is presented to participants]?
   1. Do you think it was an important part of the research?
   2. Would you want this measure to be included in future trials?
5. How did you feel about completing the Quality of Life questionnaire?
   1. Do you think it was an important part of the research?
   2. Would you want this measure to be included in future trials?
6. How did you feel about completing the Fatigue Severity Scale?
   1. Do you think it was an important part of the research?
   2. Would you want this measure to be included in future trials?
7. How easy was it to report any symptoms during exercise?
8. Did you report any serious symptoms during exercise?
   1. What happened?
   2. Did you carry on taking part in the trial?
   3. Do you think was dealt with appropriately?

Overall

1. [From pre-trial answers] – In your first interview you said you were worried about XXXX – was this worry realised when you actually took part in the trial?
2. If another patient asked you about taking part in this programme, what would you say?
3. You said in the last interview that you were hoping to achieve XXXX by taking part in the interview – do you think you achieved this?
   1. Why/why not?
4. You said in the last interview that you did/did not expect this intervention to work for you – were you right?
   1. Why/why not?
   2. Have you experienced an improvement in any of your symptoms?
5. Overall, have there been any positive impacts you have experienced by taking part in the trial?
6. Overall have there been any negative impacts you have experienced by taking part in the trial?
7. Will you continue with the at-home activities?
8. Do you have anything else you would like to add?

Post-trial (drop-outs)

1. We are really interested to find out more about why some people drop out of the study part-way through. Please could you tell me about your decision to drop out?
   1. What in particular, made it difficult for you to take part?
   2. What could we do to make it easier for people to stay in the study in future?
   3. Is there anything you would change about the study?
   4. Is there anything you would keep the same about the study?
2. [From pre-trial answers] – In your first interview you said you were worried about XXXX – was this worry realised when you actually took part in the trial?
3. If another patient asked you about taking part in this programme, what would you say?
4. You said in the last interview that you were hoping to achieve XXXX by taking part in the interview – do you think you achieved this?
   1. Why/why not?
5. You said in the last interview that you did/did not expect this intervention to work for you – were you right?
   1. Why/why not?
6. Do you have any other comments you would like to add?

Declined participation and pre-trial drop-outs

1. We are really interested to find out more about why some people would rather not take part in the study. Please could you tell me about your decision to not take part?
2. Is there anything about the study that made it more difficult for you to take part?
3. What could we have done to make it easier for you to take part?
4. What information did you read (for example, an email, or the Participant Information Sheet) about the trial before making your decision?
5. Is there anything that you would have changed about the participant information sheet or the other information you received?
6. If another patient asked you about taking part in this programme, what would you say?
7. Do you have any other comments you would like to add?
