# Supplementary material for: Exercise rehabilitation for people with postural tachycardia syndrome at two secondary care centres in the UK: the PULSE feasibility randomised controlled trial
Source: BMJ Open. 2025 Feb 22;15(2):e090197. doi: 10.1136/bmjopen-2024-090197 (PMC11848665; doi:10.1136/bmjopen-2024-090197)
Supplement: Supplementary file 2 [file bmjopen-2024-090197.reviewer_comments.pdf]

PEER REVIEW HISTORY

BMJ Open publishes all reviews undertaken for accepted manuscripts. Reviewers are asked to complete a checklist review form and are provided with free text boxes to elaborate on their assessment. These free text comments are reproduced below.

ARTICLE DETAILS

Title (Provisional)

Exercise rehabilitation for people with postural tachycardia syndrome at two secondary care centres in the UK: the PULSE feasibility randomised controlled trial.

Authors

McGregor, Gordon; Evans, Becky; Sandhu, Harbinder Kaur; Bruce, Julie; Devi, Gita; Hayat, Sajad; Hee, SiewWan; Heine, Peter; Holliday, Nikki; Joshi, Shivam; Kavi, Lesley; boon , lim; Noufaily, Angela; Parsons, Nicholas; Patel, Shilpa; Pearce, Gemma; Powell, Richard; Schultz, Eva; simmonds, jane; Zhupaj, Albiona; Eftekhari, Helen; Panikker, Sandeep

---

VERSION 1 - REVIEW

**Reviewer**	**1**
**Name**	**Gordon, Brett**
**Affiliation**	**La Trobe University, Rural Allied Health**
**Date**	**03-Oct-2024**
**COI**	**None**

The authors have reported on a study conducted to evaluate the feasibility and acceptability of a remotely supervised exercise rehabilitation program for people with POTS.

Broadly the study design is good and the reporting is adequate, but there are concerns with the data analysis approach and interpretation of data to arrive at the stated conclusions.

This is clearly stated as a feasibility trial, for which it is inappropriate to determine a sample size on data sufficient to determine a difference. It is also inappropriate to do any comparision between intervention groups due to the study not being powered to do so, yet the authors have directly compared outcomes between interventions.

The authors have also determined that the intervention was feasible and acceptable, despite not achieving the desired recruitment target and having withdrawals or dropouts greater than anticipated. This reviewer appreciates the uncertain environmental conditions (i.e. COVID-19) occuring at the time that might have influenced this. However, clear targets and outcomes should have been set a-priori for determination of feasibility and acceptability.

For this manuscript to be appropriate for publication, the following changes are suggested as required.

1. Provide clear outcomes that would be considered as representing feasible and acceptable in the data analysis plan. i.e. what proportion of screened, to enrolled was going to be considered as acceptable? What proportion of withdrawals or dropouts would be considered as representing lack of feasibility or acceptability.

2. Define adherence. This looks as though it was attendance rather than adherence, and if so, consider using this alternative term. With most participants exercising one or less times a week, was adherence actually good?

3. Define the acceptability measure/outcome. I have determined that this was made through the qualitative interviews, but it is unclear what was asked in the interviews and whether this actually determines acceptability.

4. Provide more detail and clarity about the qualitative component of the study. I note there is an intention to report this data fully in a separate manuscript, in which case it should be clearly articulated which part/s of the interview were focused on acceptability and what the focus of the remaining portion of the interview was. As it is currently reported, I do not feel as this part of the study could be replicated.

5. The data analysis section needs to be expanded to identify the criteria for determining feasibility and acceptability. It is identified in this section that a process evaluation is to be conducted, which is a good idea, but there is no detail about this and it is also the first mention of the process evaluation. How is the process evaluation being conducted and analysed? The data analysis should closely reflect the aims, and I do not believe it does at present.

7. Data should be reported for each intervention at each time point, however it should not be compared. Therefore, reporting data as a difference between interventions should not be reported (i.e Table 2).

8. Results need to be reported against the a-priori criteria for feasibility and acceptability - currently this is not the case and the criteria have not been established.

9. The discussion is likely to need revision after the data analysis and results have been re-considered.

**Reviewer**	**2**
**Name**	**GOMEZ MOYANO, ELISABETH**
**Affiliation**	**Hospital Regional Universitario de Malaga**
**Date**	**07-Nov-2024**
**COI**	**None**

It is a interesting manuscript about a debilitanting disease.

The article highlights the importance of exercise as a treatment.

The role of physical activity including exercise in severely exercise-intolerant patients remains unclear and should be undertaken with great caution.

However the authors must include more references, for example

Gómez-Moyano E, Rodríguez-Capitán J, Gaitán Román D, Reyes Bueno JA, Villalobos Sánchez A, Espíldora Hernández F, González Angulo GE, Molina Mora MJ, Thurnhofer-Hemsi K, Molina-Ramos AI, Romero-Cuevas M, Jiménez-Navarro M, Pavón-Morón FJ. Postural orthostatic tachycardia syndrome and other related dysautonomic disorders after SARS-CoV-2 infection and after COVID-19 messenger RNA vaccination. Front Neurol. 2023 Aug 16;14:1221518. doi: 10.3389/fneur.2023.1221518. PMID: 37654428; PMCID: PMC10467287.

This narrative review include 131 references.

A clincal trial with only 27 references is poor.

The keywords must include postural tachycardia syndrome, postural orthostatic tachycardia, autonomic dysfunction

**Reviewer**	**3**
**Name**	**Rocco, Patricia**
**Affiliation**	**University of Utah Health**
**Date**	**27-Dec-2024**
**COI**	**None**

The article was clear and well written. Introduction and background were reasonable with the paper premise. Literature references were sufficient. The tables are comprehensive and helpful.

The article brings a good perspective about feasibility and online exercise rehabilitation and behavioral/motivational support intervention compared to best-practice usual care for patients with POTS. There is a need of research to identify the optimal exercise rehabilitation program for this patient population.

The study is relevant and aligns with the aims and scope of the journal.

Just a few adjustments suggested:

1. To include more details about POTS diagnosis for inclusion criteria. Explore why more than a half of patients were ineligible due to did not have a confirmed POTS

diagnosis. (Line 57)

2. Explore strength and limitations. What are the reasons why the study did not have the strength to confirm the results?
3. Explore why patients did not have the time for exercise. Are they missing education about POTS? How can we change their behavior?

VERSION 1 - AUTHOR RESPONSE

Reviewer: 1

The authors have reported on a study conducted to evaluate the feasibility and acceptability of a remotely supervised exercise rehabilitation program for people with POTS.

Broadly the study design is good and the reporting is adequate, but there are concerns with the data analysis approach and interpretation of data to arrive at the stated conclusions.

This is clearly stated as a feasibility trial, for which it is inappropriate to determine a sample size on data sufficient to determine a difference. It is also inappropriate to do any comparision between intervention groups due to the study not being powered to do so, yet the authors have directly compared outcomes between interventions.

We agree that a power calculation is not required for feasibility studies. However, as stated in our prospective ISRCTN registration and protocol paper (1), the sample size was not based on a power calculation, rather, the aim was to estimate any possible effect of the change of heart rate from supine to 10-minute stand between the two trial arms using the confidence interval (CI) approach to provide a given level of precision. We are explicit throughout the manuscript that the study was not powered to detect any treatment effects.

We have removed the mean difference data calculated with the generalised linear model. We now present only mean data by treatment arm and timepoint.

The authors have also determined that the intervention was feasible and acceptable, despite not achieving the desired recruitment target and having withdrawals or dropouts greater than anticipated. This reviewer appreciates the uncertain environmental conditions (i.e. COVID-19) occuring at the time that might have influenced this. However, clear targets and outcomes should have been set a-priori for determination of feasibility and acceptability.

Please see response to point 1 below.

For this manuscript to be appropriate for publication, the following changes are suggested as required.

1. Provide clear outcomes that would be considered as representing feasible and acceptable in the data analysis plan. i.e. what proportion of screened, to enrolled was going to be considered as acceptable? What proportion of withdrawals or dropouts would be considered as representing lack of feasibility or acceptability.

A priori we chose not to set arbitrary feasibility progression criteria. This is explicitly and transparently detailed in our protocol papers(1, 2) and ISRCTN registration. The ADePT (A Process for Decision-making after Pilot and Feasibility Trials) framework (3) was used to identify and examine issues and problems methodically, and to appraise and find appropriate solutions to inform the decision-making process to transition from a two-centre feasibility trial to a definitive multi-centre RCT. In a complex clinical population and turbulent healthcare landscape many uncertainties relating to feasibility were identified and assessed. Rather than applying arbitrary cutoffs, we preferred a pragmatic approach to assessing multiple interdependent criteria that could influence the feasibility of conducting a definitive RCT. Retrospective determination of feasibility progression criteria would reduce the scientific integrity of our trial.

2. Define adherence. This looks as though it was attendance rather than adherence, and if so, consider using this alternative term. With most participants exercising one or less times a week, was adherence actually good?

Thank you for the suggestion. We have now provided a definition of adherence for this study.

We report that adherence to supervised live online exercise and support sessions was excellent (median 15 (IQR 12 to 17) of 18 sessions). We also report that unsupervised home exercise bike usage and viewing of on-demand home exercise videos was variable. So, we consider that adherence to supervised sessions was good whilst adherence to unsupervised sessions was suboptimal.

3. Define the acceptability measure/outcome. I have determined that this was made through the qualitative interviews, but it is unclear what was asked in the interviews and whether this actually determines acceptability.

We have now included the interview topic guide in supplementary material and added additional detail to the ‘embedded qualitative study’ section in the methods.

4. Provide more detail and clarity about the qualitative component of the study. I note there is an intention to report this data fully in a separate manuscript, in which case it should be clearly articulated which part/s of the interview were focused on acceptability and what the focus of the remaining portion of the interview was. As it is currently reported, I do not feel as this part of the study could be replicated.

Please see response to points 1 and 3 above.

5. The data analysis section needs to be expanded to identify the criteria for determining feasibility and acceptability.

Thank you. We have added to this section to mirror our protocol paper.

It is identified in this section that a process evaluation is to be conducted, which is a good idea, but there is no detail about this and it is also the first mention of the process evaluation. How is the process evaluation being conducted and analysed? The data analysis should closely reflect the aims, and I do not believe it does at present.

We did not conduct a full process evaluation thus have not described this on our methods section. However, as a contributor to the assessment of feasibility, we assessed ‘process

measures' (as identified in the data analysis section) for which the data are reported extensively in the results section. Process measures relating to screening, recruitment, outcome measures and intervention delivery are reported quantitatively and qualitatively.

7. Data should be reported for each intervention at each time point, however it should not be compared. Therefore, reporting data as a difference between interventions should not be reported (i.e Table 2).

Thank you we have rectified this. Table 2 now contains data by treatment arm at each timepoint with no statistical comparison.

8. Results need to be reported against the a-priori criteria for feasibility and acceptability - currently this is not the case and the criteria have not been established.

Please see response to point 1

9. The discussion is likely to need revision after the data analysis and results have been re-considered.

We have made revisions as required

Reviewer: 2

It is a interesting manuscript about a debilitanting disease.
The article highlights the importance of exercise as a treatment.
The role of physical activity including exercise in severely exercise-intolerant patients remains unclear and should be undertaken with great caution.

However the authors must include more references, for example
Gómez-Moyano E, Rodríguez-Capitán J, Gaitán Román D, Reyes Bueno JA, Villalobos Sánchez A, Espíldora Hernández F, González Angulo GE, Molina Mora MJ, Thurnhofer-Hemsi K, Molina-Ramos AI, Romero-Cuevas M, Jiménez-Navarro M, Pavón-Morón FJ. Postural orthostatic tachycardia syndrome and other related dysautonomic disorders after SARS-CoV-2 infection and after COVID-19 messenger RNA vaccination. Front Neurol. 2023 Aug 16;14:1221518. doi: 10.3389/fneur.2023.1221518. PMID: 37654428; PMCID: PMC10467287.

This narrative review include 131 references.

A clincal trial with only 27 references is poor.

We have added some references where relevant as requested. As a feasibility RCT, a very lengthy reference list is not warranted.

The keywords must include postural tachycardia syndrome, postural orthostatic tachycardia, autonomic dysfunction

Thank you for the suggestion. We have added 'autonomic dysfunction' as a key word, however it is not necessary at add 'postural tachycardia syndrome' or 'postural orthostatic tachycardia' as these words are already in the title of the manuscript so will be identified in key word search strategies accordingly.

Reviewer: 3

Comments to the Author:
The article was clear and well written. Introduction and background were reasonable with the paper premise. Literature references were sufficient. The tables are comprehensive and helpful.

The article brings a good perspective about feasibility and online exercise rehabilitation and behavioral/motivational support intervention compared to best-practice usual care for patients with POTS. There is a need of research to identify the optimal exercise rehabilitation program for this patient population.

The study is relevant and aligns with the aims and scope of the journal.

Just a few adjustments suggested:

1. To include more details about POTS diagnosis for inclusion criteria.

Thank you we have added this detail to the 'participants' section.

Explore why more than a half of patients were ineligible due to did not have a confirmed POTS diagnosis. (Line 57)

We have now provided an explanation for this in the discussion. At one study site, general cardiac arrhythmia patient lists were screened rather than an exclusively PoTS patient list.

2. Explore strength and limitations. What are the reasons why the study did not have the strength to confirm the results?

Strengths and limitations are included after the discussion section, and we have also added bullet points at the start of the manuscript as requested by the editor.

3. Explore why patients did not have the time for exercise. Are they missing education about POTS? How can we change their behavior?

This question was explored in more detail in our semi structured participant interviews. Qualitative findings will be published in full elsewhere. In the current manuscript, word count limitations prohibit a more extensive discussion of this point.

1. McGregor G, Hee SW, Eftekhari H, Holliday N, Pearce G, Sandhu H, et al. Protocol for a randomised controlled feasibility trial of exercise rehabilitation for people with postural tachycardia syndrome: the PULSE study. Pilot Feasibility Stud. 2020;6:157.
2. McGregor G, Evans B, Sandhu H, Simmonds J, Joshi S, Devi G, et al. Protocol update for a randomised controlled feasibility trial of exercise rehabilitation for people with postural tachycardia syndrome: the PULSE study. Pilot Feasibility Stud. 2022;8(1):101.
3. Bugge C, Williams B, Hagen S, Logan J, Glazener C, Pringle S, et al. A process for Decision-making after Pilot and feasibility Trials (ADePT): development following a feasibility study of a complex intervention for pelvic organ prolapse. Trials. 2013;14:353.

VERSION 2 - REVIEW

**Reviewer**	**1**
**Name**	**Gordon, Brett**
**Affiliation**	**La Trobe University, Rural Allied Health**
**Date**	**22-Jan-2025**
**COI**	

Thank you to the authors for considering and responding to my previous comments. The actions are appropriate and I think the manuscript is now appropriate for publication.

**Reviewer**	**3**
**Name**	**Rocco, Patricia**
**Affiliation**	**University of Utah Health**
**Date**	**28-Jan-2025**
**COI**	

I have read the authors' responses to my previous comments and reviewed the revised manuscript. Overall, the authors have addressed the majority of my concerns, and the revisions have strengthened the clarity and depth of the study. However, I have a few additional comments and suggestions that I believe would further enhance the manuscript:

1. On streghts and limitations section I suggest to include the reason why eight participats dropped out from the study.

2. For each limitation, describe how it may have influenced the results, and what could be improved for the future

VERSION 2 - AUTHOR RESPONSE

Reviewer: 1
Dr. Brett Gordon, La Trobe University
Comments to the Author:
Thank you to the authors for considering and responding to my previous comments. The actions are appropriate and I think the manuscript is now appropriate for publication.

Reviewer: 3
Dr. Patricia Rocco, University of Utah Health
Comments to the Author:
I have read the authors' responses to my previous comments and reviewed the revised manuscript. Overall, the authors have addressed the majority of my concerns, and the revisions have strengthened the clarity and depth of the study. However, I have a few additional comments and suggestions that I believe would further enhance the manuscript:

1. On strengths and limitations section I suggest to include the reason why eight participants dropped out from the study.

Thank you. Dropouts are carefully described in both the CONSORT diagram (figure 1) and the 'Retention' section of the results. At 4-month follow-up, 15 participants had dropped out, and at 12-months a further 4 participants had dropped out. Of the 15 patients who dropped out at 4 months, 8 were lost to follow up. We believe this may be what the reviewer is referring to. These participants were lost to follow up, so we do not have reasons unfortunately. We do not believe that the dropout rate is a limitation of the study as such, rather it is an important feasibility finding which we have discussed in the paper.

2. For each limitation, describe how it may have influenced the results, and what could be improved for the future

Amended thank you. We have added some additional text where appropriate.

VERSION 3 - REVIEW

**Reviewer**	**3**
**Name**	**Rocco, Patricia**
**Affiliation**	**University of Utah Health**
**Date**	**06-Feb-2025**
**COI**	

I appreciate the authors for considering and addressing my previous comments. The revisions are appropriate, and I believe the manuscript is now ready for publication.
